# The impact of COVID‐19 restrictions in the United Kingdom on the positive behavioural support of people with an intellectual disability

**DOI:** 10.1111/bld.12379

**Published:** 2021-02-24

**Authors:** George C. Murray, Karen McKenzie, Rachel Martin, Aja Murray

**Affiliations:** ^1^ Northumbria University Newcastle upon Tyne UK; ^2^ NHS Lothian Edinburgh UK; ^3^ University of Edinburgh Edinburgh UK

**Keywords:** COVID‐19, intellectual disability, positive behavioural support, social care staff

## Abstract

- The way that people with an intellectual disability are supported is very important.
- The COVID‐19 virus has changed the way that staff help people with an intellectual disability.
- We wanted to know about those changes and whether learning about positive behavioural support (PBS) helped staff to cope with them.
- The main changes were that people with an intellectual disability could not go out or see family and friends as often.
- Staff came up with new things to do for the people they supported, and PBS learning seemed to help staff to cope.

**Abstract:**

**Background:**

It has been suggested that COVID‐19 and the associated restrictions are likely to have a negative impact on the provision of positive behavioural support (PBS) to people with an intellectual disability.

**Methods:**

Fifty‐eight staff, who had recently completed an accredited positive behavioural support (PBS) programme, responded to an online questionnaire, which asked them to rate the impact of COVID‐19 on factors related to PBS.

**Results:**

Participants reported a neutral or somewhat positive impact on all the areas measured, with the exception of the activities and quality of life of those they supported, which were somewhat negatively affected. The participants rated the learning from their PBS programme as helping them cope with COVID‐19 to some extent. Examples of positive and negative effects and ways in which PBS helped staff to cope are presented.

**Conclusions:**

Many staff developed creative solutions that allowed them to provide PBS despite the COVID‐19 restrictions. PBS learning appeared to help staff cope with the negative impact of the restrictions.

## 1 INTRODUCTION

As a result of the COVID‐19 pandemic, restrictions have been introduced in many countries across the world in an attempt to reduce the spread of the disease. The “lockdown” restrictions in the United Kingdom (UK) began in March 2020. While there were some differences between the four nations, the initial restrictions broadly required people to maintain social distance from, and restrict contact with, others, stay at home as much as possible and “shield” if they were part of a vulnerable group. All but essential shops and activities were closed.

Many people with an intellectual disability are potentially particularly vulnerable to the effects of the COVID‐19 pandemic because of existing healthcare needs, such as respiratory disease, that place them at increased risk of more severe outcomes (Turk et al., 2020). The restrictions are also likely to impact significantly on their quality of life because many rely on the support of others to help them to access community activities, socialise and structure their day (Courtenay & Perera, 2020).

These elements and others have long been highlighted as important components in high‐quality care and are captured in O'Brien's (1992) Five Accomplishments Framework. Here, “Relationships” refers to supporting people to have positive and meaningful relationships. “Competence” relates to promoting the development of skills and engagement in meaningful activities, while “Choice” refers to encouraging the ability to make decisions and express preferences. “Community Presence” reflects active participation in community activities and resources. Finally, “Respect” reflects the right of people with an intellectual disability to be treated in valued and respectful ways and to be supported to avoid behaviours that would lead to them being viewed negatively.

Some people with an intellectual disability do, however, display behaviours that challenge, which are often viewed negatively by others (Jahoda & Wanless, 2005), with prevalence rates estimated as being 18% in those who are known to services (Bowring et al., 2019). Positive behavioural support (PBS) offers a functional, constructional and values‐based approach to such behaviours. PBS has three key components: values, theory and evidence base, and process (Gore et al., 2013). The first relates to the need to view behaviours that challenge in the context of the person's overall quality of life and to reduce the need for such behaviours by improving quality of life. The second refers to the use of behaviour analysis approaches to gain an understanding of the function of the behaviour. This informs the initial development of evidence‐based strategies to support behaviour change. The final component refers to the processes involved in developing function‐based, multicomponent interventions (often referred to as PBS plans), which use proactive and reactive approaches to modify and manage the behaviour, respectively.

It has been suggested that staff may have difficulty in implementing existing PBS plans because of COVID‐19 and the associated restrictions, and that disruption to the activities and routines of people with an intellectual disability present the potential for increased behaviours that challenge (Courtenay & Perera, 2020). There has only been limited research into the impact of COVID‐19 on social care staff and on behaviours that challenge. In terms of the latter, one study in the Netherlands found that, after an initial drop in reporting of incidents of aggression at the start of COVID‐19 restrictions, levels increased significantly (Schuengel et al., 2020).

In respect of the impact on social care staff, Embregts et al. (2020) conducted a small qualitative study with 11 care staff in the Netherlands and found that the staff reported both positive and negative effects of COVID‐19. They had concerns about becoming infected and expressed frustration that their role and the vulnerable position of people with an intellectual disability were largely overlooked. They used a range of coping strategies including a focus on the job at hand, discussions with colleagues and reflection. Staff also generated creative solutions to try to overcome the negative impact of the restrictions.

The current study aimed to add to this limited body of research. Here, we explore the impact of the COVID‐19 restrictions on social care staff working in intellectual disability services, in the specific context of PBS approaches. We aim to address the following related questions:

- What is the impact (both positive and negative) of COVID‐19 restrictions on key elements of PBS approaches, as reported by social care staff?
- In what ways do staff perceive PBS input as helping them cope with the COVID‐19 restrictions?

## 2 METHOD

### 2.1 Design

The study used an observational design, and data were collected via an online survey. The study was an addition to an existing evaluation of the impact of a PBS programme. An amendment was submitted to cover this addition and was granted by the second author's university ethics board.

### 2.2 Participants

Overall, 58 people took part, of whom 17 (29.3%) were male and 41 (70.7%) were female. All but two participants were White. Ages ranged from 23 to 63 years (*M* = 43.1, *SD* =10.5). All participants were staff working in social care settings who supported people with an intellectual disability. Seventeen (38.6%) were managers, 25 (56.8%) were support workers, and 2 (4.5%) had another role (missing data =14).

The participants were part of a wider study that was evaluating the impact of a regional PBS approach in the North East of England. The participants had all recently completed an accredited programme in PBS practice, the level of which depended on their specific role. Each programme comprised two or three modules, and each module lasted for three months. The input was a mixture of face‐to‐face and online teaching, and workplace support and supervision were provided. The content included the topics of valuing people with an intellectual disability; gathering and using data; functional behavioural analysis; and behaviour skills training (see McKenzie, Martin, et al., 2020 for details). The programme was one aspect of a region‐wide workforce development approach, which aimed to improve the PBS practice of staff, with the ultimate goals of improving the quality of life and reducing the behaviours that challenge of the people with an intellectual disability whom they supported (McKenzie, McNall, et al., 2020).

Inclusion criteria were that the participants were aged 18 or above, were participating in the wider study and had provided informed consent.

### 2.3 Measures

Participants provided basic demographic information before completing the measures below.

#### 2.3.1 The impact of COVID‐19 on PBS‐related elements

Participants were asked to respond to the question: “To what extent has the coronavirus impacted on the following?” in relation to the areas outlined in Table 1. These areas have been found in previous research to be important in the context of PBS approaches, such as developing and implementing PBS plans, staff confidence in managing behaviours that challenge and applying knowledge in practice (e.g., MacDonald et al., 2018; McKenzie, Martin, et al., 2020). Ratings were from 1 = very negative to 5 = very positive. Participants were also asked to give examples of the main positive and negative impacts of COVID‐19 on their ability to provide PBS to the main person they supported.

**TABLE 1:** Mean scores and standard deviations in relation to the impact of COVID‐19 and the associated restrictions on staff and those they supported

Area of impact	Mean (SD)
Applying knowledge of PBS in practice	3.0 (1.1)
Carrying out the behaviour support plan of the main person you support	2.8 (1.1)
Feeling confident in effectively managing behaviours that challenge	3.6 (1.0)
The activities of the main person you support	2.3 (1.3)
The behaviours that challenge of the main person you support	2.6 (1.1)
The quality of life of the main person you support	2.4 (1.3)

Note:

A higher score indicates a more positive impact (possible range 1–5)

#### 2.3.2 Coping score

Participants were asked to rate the extent to which they considered that learning from the PBS programme had helped them cope with the restrictions related to COVID‐19. Ratings were from 1 = not at all to 3 = to a large extent. They were then asked to give an example (if applicable) of the main way that the PBS learning had helped them to cope.

### 2.4 Analysis

Descriptive statistics were used to present the quantitative data. Qualitative responses were analysed using directed content analysis (Hsieh & Shannon, 2005). This method was chosen to enable a structured analysis, which was informed by existing frameworks, in this case the Five Accomplishments Framework (O'Brien, 1992) for the “impact” responses, and the PBS framework (Gore et al., 2013) for the “coping” responses. Brief operational definitions were developed from these frameworks, which guided the coding of the text responses. Responses that did not fit within the categories were coded as “other.” These largely related to participants reporting no positive impact of COVID‐19, (e.g. “none,” “nothing”) and are not reported in the results. Example responses were then chosen to illustrate each coding category. The data that support the findings of this study are available from the corresponding author upon reasonable request.

### 2.5 Procedure

Participants were contacted via existing contact details and were invited to take part. Data were gathered via an online questionnaire between April and May 2020, at the height of the COVID‐19 restrictions in the UK. This questionnaire provided information about the study, and participants confirmed consent before going on to complete the measures. Responses were anonymous, with participants providing their own codes.

## 3 RESULTS

### 3.1 The impact of COVID‐19

Table 1 illustrates the mean scores in relation to each of the “impact” areas measured. This shows that, on average, the COVID‐19 restrictions had a neutral or somewhat positive impact on all but two areas: the activities and quality of life of the person that the participant supported.

### 3.2 Positive and negative impacts of Covid‐19 restrictions

Table 2 provides examples of the positive and negative impacts of COVID‐19 on participants' ability to provide PBS, coded according to the Five Accomplishments Framework (O'Brien, 1992). The negative aspects commonly related to restricted activities and social contact of the person being supported. The positive aspects reflected staff creativity in finding solutions and the positive use of the extra time they spent with those they supported.

**TABLE 2:** The most commonly cited examples of the positive and negative impacts of COVID‐19 on participants' ability to provide PBS to the main person they supported, presented within the Five Accomplishments Framework (O'Brien, 1992).

	Negative	Positive
Category	Example responses	
Community presence		
Supporting activities and community presence	The impact on social, community presence. Lack of external activities for someone who loves external activities. Limited activities in community.	Being more creative in the activities we do daily to support quality of life. It has forced us to become more creative with the resources we have, to be able to meet the individuals' needs. Everyone has pulled together. People willing to try things to adapt plan to circumstances.
Relationships		
Relationships with family, friends and staff	Can't see his dad. Visiting family (unable to) Limiting social contact and activities.	Developing closer relationship. Give the individual all your time and attention. More engagement and time spent with staff
Choice		
Promoting choice	It has taken away some choices—such as preferred external activities. [Challenging] behaviours due to not doing the activities they have always chosen and like to do. Unable to complete preferred activities away from home.	More time has been able to be allocated to individuals accessing the service which has enhanced their ability to make choices and decisions, reducing behaviours of concern. It has given us time to spend working on his care plan which is something he enjoys
Competence		
Opportunities to promote skills and competence	Not allowed to attend work. The ability to develop individual social ability within community.	Teaching new skills—social distancing. Introducing new skills in the home environment. Able to develop skills within home life.
Respect		
Opportunities to provide valued identity and provide support in positive ways	Has meant that the client hasn't had the 1:1 staff he is used too and consequently replacement staff not as comfortable and familiar with plan, therefore leading to some increases in behaviour that challenges. Behaviours due to not doing the activities they have always chosen and like to do	Knowing the person and being proactive in our approach to the changes required. More active support! We have started to support service user in the community… to prevent family breakdown and this has given staff the opportunity to reintroduce PBS techniques with him

### 3.3 PBS learning and coping

Of those who responded, 16 (40%) felt their PBS learning had helped them to cope with the COVID‐19 restrictions to a large extent, 17 (42.5%) to some extent and 7 (17.5%) felt it had not helped at all. Table 3 illustrates examples of the ways in which PBS learning helped participants to cope with the negative impact of COVID‐19 and the associated restrictions, coded according to the key components of PBS (Gore et al., 2013). These include helping staff to provide support in positive, constructive ways that improve quality of life; understand the functional nature of behaviours that challenge; and use this understanding and other evidence to inform interventions.

**TABLE 3:** Examples of the ways in which PBS learning helped participants to cope with the negative impact of COVID‐19 and the associated restrictions, presented within the context of key components of PBS (adapted from Gore et al., 2013).

Key component of PBS	Related constructs illustrated in the examples	Example responses
Values	Recognising the needs of, and supporting individuals (including staff) in positive, constructive ways that improve their quality of life. Behaviours that challenge are seen in this wider context.	Spending quality time with the individual. By speaking to service users daily, I am able to offer support and reassurance over the phone. Helped me support staff to think outside the box in supporting individuals. Have had to be flexible to ensure service user remains settled and continues to have a good quality of life, even if different than before. [It has given] the customer I care for choices and learning new skills. It has helped in the way I support staff with their wellbeing. A better understanding of the impact on the service user and how to effectively help with the change of routine. The additional thinking and reflection on behaviour and ability to impact on this positively.
Theory and evidence base	Understanding the functional nature of behaviours that challenge.	Made me look at functions of behaviour and has helped me to understand that when customers are restricted in their everyday life, this can have a negative impact on their behaviours. More likely to look at the cause behind the behaviour. I've had to think more quickly about functions of behaviour. The PBS course has given me a useful knowledge to know that I am dealing with behaviours that challenge correctly. The learning has given me knowledge to relay to the staff team which has improved the way we interact and support individuals.
Process	Using evidence and functional understanding of behaviour to inform interventions.	Allowed me to think creatively and using current evidence of behaviour of the person. By having a clear understanding of the functions of each individual's behaviours and a good understanding of what interventions that are successful. This has made it a lot easier to tailor support plans to factor in the difficulties that the restrictions linked to covid have brought. PBS has helped me understand the functions of individuals' behaviours allowing me to explore different ways to meet their needs throughout the epidemic. Implementing proactive and preventative strategies, forward thinking, working closely with family and friends as well the customer teams to think of alternative ways to manage the restrictions, ensuring the quality of life of the individual is at the forefront. Made me think about engagement with customer at 20‐minute intervals to stop them becoming withdrawn or bored.

## 4 DISCUSSION

In the light of the significant restrictions imposed as a result of the COVID‐19 pandemic, the aim of the study was to explore staff reports of the perceived impact, in the specific context of providing PBS. The areas that were included have been found by researchers to be important in the context of PBS approaches, such as applying PBS knowledge in practice, developing PBS plans, implementing them in practice, and managing any behaviours that do occur with confidence (e.g. MacDonald et al., 2018; McKenzie, Martin, et al., 2020). As PBS aims to remove the need for the person to display behaviours that challenge, by providing functional alternatives, meaningful activities and improved quality of life (PBS Coalition UK, 2015), the impact on these areas was also measured. The results illustrated that COVID‐19 and the associated restrictions were reported as having had a largely neutral or somewhat positive impact on most of the areas measured, with the exception of the activities and quality of life of the main person that the participant supported, which were seen as being more negatively affected.

When considering the examples of both positive and negative impacts of the COVID‐19 restrictions, these could be situated within the Five Accomplishments Framework (O'Brien, 1992). The most common negative aspects were restricted activities and social contact. These most obviously affected the community presence and relationships of the people being supported, but also reduced their choices, and their opportunities to demonstrate competence through existing skills and work. The reported negative impact on behaviours that challenge also influenced the area of respect; as such, behaviours can lead people with an intellectual disability to be viewed more negatively (Jahoda & Wanless, 2005).

In terms of positive aspects, the staff used the changing situation to create new opportunities for skills teaching, at times explicitly in relation to COVID‐19, such as how to maintain the required social distance from others. While external activities were generally curtailed, the staff were creative in developing alternatives that were based in and around the home. The increased time spent with the individuals they supported provided the opportunity for more tailored, individualised support, a deeper understanding of the person and closer relationships. This extra time also afforded the chance to support the person to express their preferences and increase control over the range of choices that were available to them. The need for social care staff to be creative and problem solve in the face of COVID‐19 restrictions was also highlighted in the research by Embregts et al. (2020).

Overall, 82.5% of participants indicated that the PBS learning had helped them to cope with the COVID‐19 restrictions to a large or to some extent. PBS training has previously been associated with increases in staff confidence and self‐efficacy (see MacDonald et al., 2018). It may be that the PBS input helped participants in the present study to feel more confident and in control, despite the difficult circumstances they faced. Ways in which PBS learning had helped them to cope in practice were located within the three overarching components of PBS. In terms of “values,” positive, person‐centred support was provided that aimed to reduce stress and enhance quality of life as much as possible in the circumstances. “Theory and evidence base” was largely exemplified by the recognition that any behaviours that challenge served a purpose for the person, allowing staff to assess the function more quickly. “Process” was demonstrated by the ways in which this functional understanding was used to develop multicomponent interventions to address the person's needs.

The study did have limitations. The sample size was relatively small, and the study was based on self‐report rather than on observation of practice. In addition, the methodology did not allow for an in‐depth exploration of the impact of, or methods of coping with, the COVID‐19 restrictions in the context of PBS approaches. Further research using interviews to obtain more detailed information would help to address this latter limitation. The study also focused on the perspectives of staff. This highlights the need for future research to obtain the views of people with an intellectual disability.

In conclusion, the study suggests that many staff were able to provide PBS despite the restrictions. The participants were able to identify many positive consequences of the COVID‐19 restrictions that could be conceptualised within the Five Accomplishments Framework (O'Brien, 1992). Many participants were also able to use their PBS learning to create practical, evidence‐based solutions to address the challenges posed by the restrictions. Overall, the results suggest that PBS learning went some way to help the majority of staff cope with the impact of COVID‐19 restrictions on their ability to support people with an intellectual disability.

## CONFLICTS OF INTEREST

None.

## Data Availability

The data that support the findings of this study are available from the corresponding author upon reasonable request.
